# 

**Published:** 1886-07

**Authors:** 


CONTENTS.

VOL. 33 No. 7

| | PAGE. |
|---|---|
| Our Prospects | 193 |
| Animal Magnetism | 193 |
| Presentiments | 196 |
| Delicate Girls | 197 |
| Smoking and Drinking | 198 |
| Stragglers on the March | 199 |
| Causes and Cure of Disease | 200 |
| Keep the Sick Clean and Cool | 203 |
| Poisons Formed from Food | 204 |
| No Cure, No Pay | 205 |
| The Cigarette | 206 |
| The Evolution of Sugar | 208 |
| Medicine in China | 209 |
| What is a disease? | 211 |
| Tonga for Neuralgia | 212 |
| New Method with Dahlias | 214 |
| Iodide of Potassium in Asthma | 215 |
| Curiosities of Digestion | 215 |
| One Hundred Glasses a Day | 216 |
| A Child with Rabbits' eyes | 217 |
| Awoke in his Coffin | 217 |

ADVERTISEMENTS OF HALF A PAGE AND OVER.

| | PAGE. |
|---|---|
| Fellows' Hypo-Phos-Phites | I |
| Wells, Richardson & Co's Lactated Food | II |
| Celerina | III |
| Lactopeptine | IV |
| Castoria | IV |
| Hosford's Acid Phosphate | V |
| Caulocorea | V |
| Medicated Suppositories | VIII |
| Orange Blossom | 2d page of cover |
| Perforated Paper | 3d page of cover |
| Lotta and Her Mother | 4th p. cover |

